# Effectiveness of the BOOST-A™ online transition planning program for adolescents on the autism spectrum: a quasi-randomized controlled trial

**DOI:** 10.1186/s13034-017-0191-2

**Published:** 2017-10-10

**Authors:** Megan Hatfield, Marita Falkmer, Torbjorn Falkmer, Marina Ciccarelli

**Affiliations:** 1 School of Occupational Therapy and Social Work, Curtin University, Perth, Australia; 2 Cooperative Research Centre for Living with Autism (Autism CRC), Long Pocket, Brisbane, QLD Australia; 3 School of Education and Communication, Institution of Disability Research, Jönköping University, Jönköping, Sweden; 4 Department of Medicine and Health Sciences (IHM), Linköping University and Pain and Rehabilitation Centre, Linköping, Sweden

**Keywords:** Asperger’s syndrome, Autism spectrum disorder, Disability, Employment, High school, Post-secondary education, Self-determination theory, Strengths-based, Career development

## Abstract

**Background:**

The majority of existing transition planning programs are focused on people with a disability in general and may not meet the specific need of adolescents on the autism spectrum. In addition, these interventions focus on specific skills (e.g. job readiness or self-determination) rather than the overall transition planning process and there are methodological limitations to many of the studies determining their effectiveness. The Better OutcOmes & Successful Transitions for Autism (BOOST-A™) is an online program that supports adolescents on the autism spectrum to prepare for leaving school. This study aimed to determine the effectiveness of the BOOST-A™ in enhancing self-determination.

**Methods:**

A quasi-randomized controlled trial was conducted with adolescents on the autism spectrum enrolled in years 8 to 11 in Australian schools (N = 94). Participants had to have basic computer skills and the ability to write at a year 5 reading level. Participants were allocated to a control (n = 45) or intervention (n = 49) group and participants were blinded to the trial hypothesis. The intervention group used the BOOST-A™ for 12 months, while the control group participated in regular practice. Outcomes included self-determination, career planning and exploration, quality of life, environmental support and domain specific self-determination. Data were collected from parents and adolescents.

**Results:**

There were no significant differences in overall self-determination between groups. Results indicated significant differences in favor of the intervention group in three areas: opportunity for self-determination at home as reported by parents; career exploration as reported by parents and adolescents; and transition-specific self-determination as reported by parents.

**Conclusions:**

Results provide preliminary evidence that the BOOST-A™ can enhance some career-readiness outcomes. Lack of significant outcomes related to self-determination at school and career planning may be due to the lack of face-to-face training and parents being the primary contacts in the study. Further research is needed to determine effectiveness of the BOOST-A™ related to post-secondary education and employment.

*Trial registration* #ACTRN12615000119594

## Background

### Post-school transition for adolescents with autism

As adolescents transition out of secondary school to adult life, they engage in a number of new roles including employment, post-secondary education, expanded community involvement, and home maintenance [1]. This transition out of high school can be particularly difficult for adolescents on the autism spectrum for a number of reasons. A key feature of autism is difficulty coping with uncertainty, which is linked to increased levels of anxiety [2]. The period of transition out of secondary school can be particularly anxiety provoking for adolescents on the autism spectrum as they face the insecurity that accompanies changing life roles [3]. Adolescents on the spectrum face unique social and communication challenges during the transition out of school [4], and difficulties often arise with managing increasing social demands, rather than challenges with actual task performance [5, 6]. Adolescents on the autism spectrum experience poorer quality of life than people without a diagnosis of autism [7] and have poorer post-school outcomes in the areas of employment and post-secondary education than adolescents with other types of disabilities as well as people without disability [8]. This suggests that existing transition planning processes may not be meeting the needs of adolescents on the autism spectrum.

### Transition planning interventions

Transition planning can support adolescents with autism to navigate the shift in roles and to prepare for leaving school [9]. Transition planning involves exploring potential careers, setting goals, and engaging in new experiences [10]. Most existing transition planning interventions target adolescents with a disability in general. A meta-analysis of interventions that taught self-determination skills to adolescents with disabilities identified 22 studies that were targeted at adolescents with intellectual disability (ID) and learning disabilities, and highlighted the need for autism-specific interventions [11]. A literature review identified 12 quantitative studies of interventions that aimed to enhance student participation in individualized education program (IEP) meetings [12]. All interventions targeted adolescents with a disability in general. The review identified that all 12 studies reported increases in either student participation in IEP meetings or increased self-determination; for example, the Whose Future Is It Anyway? program enhanced self-determination in a randomized controlled trial (N = 493) [13]. However, the authors of the review concluded that there is a need for transition planning programs that include parents in the transition planning process and that impact the adolescents’ everyday lives. Another systematic review of transition planning interventions for adolescents with disabilities in general identified that research in this area is predominantly qualitative [9]. In the existing quantitative studies, a lack of methodological rigour was identified, including use of pre-test/post-test design and no control group. For example, an evaluation of the MY VOICE program found participants were satisfied with the program but the study had no control group and used retrospective pre-testing [14]. The results of a randomized controlled trial of the Whose Future Is It Anyway? teacher-led program favored the intervention group [13], with significant between-group differences in self-determination. However, the sample included people with disabilities in general and the study did not address autism-specific needs for transition planning.

A few autism-specific transition planning studies have been published recently. One study aimed to describe important elements of effective transition planning for adolescents on the autism spectrum [15] but much of the reviewed literature was not autism-specific and findings were based on studies of people with disabilities in general. A systematic review of interventions to support transition planning for adolescents on the autism spectrum found no studies that met the inclusion criteria of quantitative research that focused on employment as an outcome, and therefore the review described qualitative research that explored transition planning for this group [16]. The authors of the review concluded that further research utilizing rigorous designs was needed to determine the effectiveness of transition planning programs for adolescents with autism.

An evaluation of an autism-specific transition planning program, Putting Feet on My Dreams, reported increased goal-directed behavior [17], but findings should be interpreted with caution due to small sample size, no control group, and use of interviews to determine the effectiveness. The results of a randomized controlled trial evaluating an autism-specific transition program found a significant between-group difference in favor of the intervention for vocational decision making ability, expectations for the future, and self-determination at year 1 [18]. However, this difference was not maintained by year 2 and the small sample size (n = 47) introduced a threat to external validity.

In summary, most existing transition planning programs were not autism-specific and the studies that determined their efficacy had methodological limitations. Most programs were developed in the United States of America and are not validated in an Australian context. This is important because of differences between countries in legislation, funding models, and service provision methods. Therefore, there is a need for a rigorously developed and evaluated autism-specific transition planning program for Australian adolescents. The Better OutcOmes & Successful Transitions for Autism (BOOST-A™) program was developed to address this need. The BOOST-A™ is an online autism-specific program developed for an Australian context that aims to prepare adolescents on the autism spectrum for leaving school. The BOOST-A™ was developed for adolescents on the autism spectrum without an ID because studies have shown that this group often have poorer outcomes than adolescents with ID because of lack of access to transition support and services [19, 20].

### Aims

The primary aim of the trial was to determine the effectiveness of the BOOST-A™ in improving self-determination among adolescents on the autism spectrum. The secondary aim was to determine the program’s impact on quality of life; access to environmental supports; career planning and exploration; and domain-specific self-determination among adolescents on the autism spectrum.

## Methods

The effectiveness of the BOOST-A™ was determined in a quasi-randomized controlled trial, in which outcomes for the intervention group (BOOST-A™) were compared to the control group (regular transition planning practice). The trial was a cluster group, two-arm, superiority trial with 1:1 allocation ratio. The full details of the study protocol have been published elsewhere [21].

### Participants

Participants were recruited between June and November 2015 via community organisations for people on the autism spectrum. A recruitment flyer was distributed on websites, social media, in person, and through email. Inclusion criteria for participants included:

- Formal diagnosis of Autism Spectrum Disorder, as defined by the Diagnostic and Statistical Manual of Mental Disorders, DSM-IV [22] or DSM-5 [23];
- Living in Australia;
- Enrolled in years 8 to 11 at school; and
- Ability to write at a year 5 reading level and possess basic computer skills.

Adolescents were excluded from the study if they had a diagnosis of ID or if they were currently enrolled in another transition planning program. Statistical power calculations indicated a minimum total sample of N = 80 (n = 40 in each group) was required to detect a standardized difference of 0.6 (Cohen’s *d*) [24], with a critical alpha of 0.05 and power of 80%.

### Intervention

The BOOST-A™ is an online program that aims to support adolescents on the autism spectrum with their transition from high school. The development of the BOOST-A™ was guided by the PRECEDE-PROCEED model [25]. A needs assessment was completed, which resulted in the development of transition planning objectives for adolescents on the autism spectrum [26, 27]. The objectives were comprised of three guiding ideals and five strategies that directed the development of the BOOST-A™. Furthermore, based on the needs assessment, three main frameworks were chosen to underpin the BOOST-A™: the self-determination model [28, 29], a strengths-based approach [30, 31], and a technology-based approach [32]. The BOOST-A™ was piloted in two studies by adolescents on the autism spectrum, their parents, educators, and allied health professionals [33], who confirmed the program was appropriate, usable, and feasible. Feedback from the pilot studies was used to modify the BOOST-A™ to enhance usability.

The BOOST-A™ consists of four modules (shown in Table 1) delivered via a website that is accessed by an individual login. The BOOST-A™ has a number of features that make it unique and autism-specific. These include provision of a clear process that supports the adolescent’s preference for structure and routine, consideration of sensory preferences and learning styles, and the inclusion of a number of animated videos that help the adolescent to understand the purpose of each module. Adherence to the intervention was monitored using website analytics; i.e., number of logins, number of modules completed, and feedback from participants about the number of times they met with the team.

**Table 1:** Overview of the BOOST-A™ transition planning program

Module	Description
1. About me	Adolescents completed six activities to identify their interests, strengths, work preferences, life skills, training goals, and learning style
2. My team	Adolescents and parents identified a team of people to support their transition planning, and then booked the first meeting. Adolescents selected their level of involvement in team meetings
3. First meeting	The team met to review career options and formulate goals, based on best-practice recommendations that are built into the program
4. My progress	The team met once per school term following the first meeting to review goal progression and positive learning experiences

The control group partook in the regular practice at their respective schools. This may have included any generic transition planning processes utilized at the school but did not include any structured or disability-specific transition planning programs. Participants in the control group were given access to the BOOST-A™ at the conclusion of the study.

### Procedures

Participants who expressed an interest to be in the study were screened for eligibility and sent a participant information sheet and consent forms. Because the adolescents were under 18 years of age, they provided written informed assent, and their parents provided written informed consent for the adolescent’s participation and their own. For the intervention group, school principals provided informed written approval for school staff to use the BOOST-A™ with the adolescents. Consent was not required from individual teachers because no data were collected from them during the study. Participants were allocated to the intervention or control group upon enrolment to the study using an alternate allocation method. The first participant was allocated to a group based on a coin toss that was completed by a researcher who was not in contact with the participants, and the second enrolled participant was allocated to the other group, and so on. The exception to this was when a new participant was attending the same school that a currently enrolled participant attended. In this case, the newly enrolled participant was allocated to the same treatment group as the currently enrolled participant. The aim of this allocation scheme was to reduce the risk of contamination, since school staff were involved in the administration of the BOOST-A™. The trial commenced on 26 November 2015 (Time point 1, T1), and post-measures were completed within 2 months of 26 November 2016 (Time point 2, T2). The 12 month timeframe was chosen to allow participants adequate time to complete the multiple modules of the BOOST-A™ program. Given the outcome measures were online, there was a 2 month period in which participants completed the outcome measures at the T2 measurement point. This could have resulted in some participants having slightly longer than 12 months to complete the BOOST-A™. Therefore, dosage was measured by the number of modules completed and the number of logins to the program.

### Outcomes

Demographic information was collected at baseline for all participants. Socio-economic status of participants was determined by Socio-Economic Indexes for Areas (SEIFA) deciles, utilising the Commonwealth Department of Education, Employment, and Workplace Relations’ measure of relative socio-economic advantage and disadvantage [34]. Data from self-reported outcome measures were collected twice: once at baseline (T1) and once 12 months later (T2). The Social Responsiveness Scale–Second Edition (SRS-2) [35] was used to classify autism severity based on a raw cut-off score of 57 [36]. Detailed information about the outcome measures and their psychometric properties was previously published in a study protocol paper [21].

The primary outcome of this trial was self-determination, as measured by the AIR Self-Determination Scale (AIR) [37]. The AIR has good test–retest reliability, internal consistency, and construct validity [37], as well as demonstrated sensitivity to change [38, 39]. There were four secondary outcomes. Career planning and exploration was measured by the Career Development Inventory—Australia—Short Form (CDI-A) [40]. The CDI-A has been found to have adequate internal consistency, concurrent validity, and construct validity [41]. Quality of life was measured by the Personal Wellbeing Index-School Children (PWI-SC) [42]. The PWI-SC has high internal consistency and construct validity [43] and demonstrated sensitivity to change [44]. Environmental support was measured by the Learning Climate Questionnaire (LCQ) [45], which has been found to have good construct validity and high internal consistency. The final outcome was domain specific self-determination, measured by the Transition Planning Objectives Scale, which was designed for this trial to evaluate the transition planning objectives identified in the needs assessment.

### Statistical analysis

The Kolmogorov–Smirnov test was used to determine normality of the data. To determine the effectiveness of the BOOST-A™ 12 months after the intervention (T2), the change in each outcome from T1 and T2 for each participant was calculated. Then the changes between intervention and control groups were compared using the independent samples t test and/or Mann–Whitney U test. There were departures from normality in several of the outcomes, so both parametric and non-parametric tests were used to compare the outcomes for participants in the intervention and control groups at baseline (T1). Results were reported using parametric statistics because analyses revealed that the parametric and non-parametric tests produced consistent results. An intention-to-treat approach was used so that participants’ data were analyzed according to the original group they were allocated regardless of actual treatment received. For participants who did not provide outcome data at T2, the last observation carried forward method was used, in which it was assumed that no change occurred in these outcomes from T1 to T2. In order to reduce the chance of a Type I error associated with conducting t-tests on the different outcomes, a multivariate analysis of variance was also conducted (implemented as a random effects regression model). In this analysis the respondent was classified as a random effect, the question number and group (intervention or control) were the independent variables, and the change in score on each question was the dependent variable. Outcomes included for analysis in this model were those that appeared to be significant through univariate analyses. The Statistical Package for the Social Sciences (SPSS v.22) [46] was used to analyze the data and a p value < 0.05 was taken to indicate a statistically significant difference in all tests. Any differences in baseline characteristics between the intervention and control groups were taken into account using a general linear model.

### Ethics

The trial received ethics approval from Curtin University Human Research Ethics Committee (approval number HR110/2014), and the Departments of Education and Catholic Education Offices in New South Wales, Western Australia, Victoria, Queensland, South Australia, and Tasmania. The trial adhered to the Australian Code for the Responsible Conduct of Research [47] and the National Statement on Ethical Conduct in Human Research [48]. The trial was also registered with the Australia and New Zealand Clinical Trial Registry (#ACTRN12615000119594) and was developed in accordance with the Consolidated Standards of Reporting Trials (CONSORT) 2010 guidelines [49].

## Results

### Participants

**Fig. 1:**
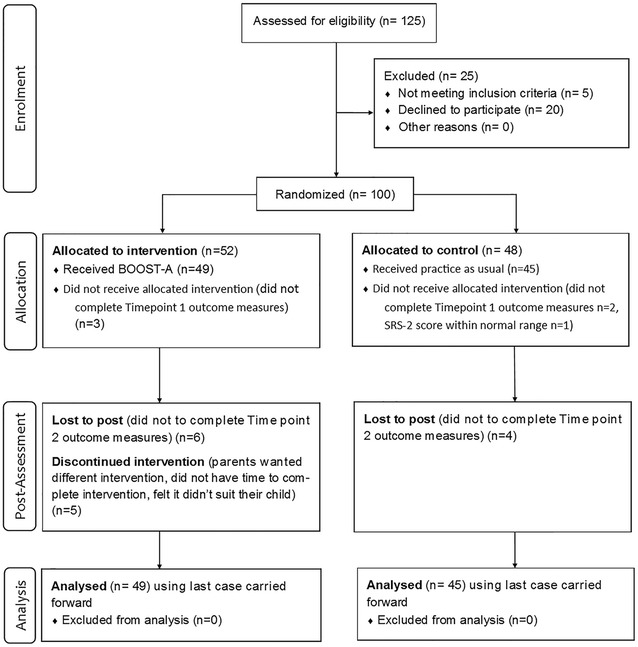
Flowchart of the BOOST-A effectiveness study

Of the 125 participants who expressed an interest in participating in the study, 100 met the inclusion criteria and enrolled in the study. A number of participants did not complete the baseline (T1) outcome measures (n = 3 in the intervention group, n = 2 in the control group) and the data from one participant in the control group were withdrawn because the SRS-2 score was within the normal range. This resulted in 49 participants in the intervention group and 45 in the control group (N = 94). The sampling procedure and the participant dropout rate can be seen in the CONSORT diagram in Fig. 1.

Baseline data reported in Table 2 show that participants were mostly male (intervention 79.6%; control 71.7%), and the average ages of the adolescents in the intervention and control groups were 14.8 and 15.1 years, respectively. SEIFA deciles range from 1 to 10; where 1 indicates the participant’s residential area is within the lowest 10% socio-economic advantage and 10 indicates the participant resides in an area within the highest 10%. The average SEIFA was 7.4 for the intervention group and 5.8 for the control group. Autism severity ranged from mild to severe in both groups. A number of participants had comorbid diagnoses; the two most common being attention deficit hyperactivity disorder intervention 10.2%; control 20.0%) and anxiety (intervention 10.2%; control 11.1%).

**Table 2:** Participant demographics by group

Pre-intervention	Group (N = 94)		*p*
	Intervention n = 49	Control n = 45	
Adolescent age in years (mean, range, SD)	14.8 (12–17, 1.2)	15.1 (13–18, 1.2)	0.215
Adolescent gender (#, %)			
Female	10 (20.4)	12 (26.7)	0.479
Male	39 (79.6)	33 (73.3)	
Socioeconomic status (SEIFA mean, range, SD)	7.4 (4–10, 2.0)	5.8 (1–10, 2.5)	0.001*
Autism severity (n, %)			
Within normal limits	0 (0)	0 (0)	0.662
Mild	5 (10.2)	5 (11.1)	
Moderate	13 (26.5)	16 (35.6)	
Severe	31 (63.3)	24 (53.3)	
Comorbid diagnoses (n, %)			
Attention deficit hyperactivity disorder	7 (14.3)	10 (22.2)	0.318
Anxiety	5 (10.2)	5 (11.1)	0.887
Dyslexia	1 (2.0)	2 (4.4)	0.508
Depression	2 (4.1)	2 (4.4)	0.931
Other	7 (14.3)	8 (17.8)	0.644

* Significant difference between Intervention and Control Group; p < 0.05

### Baseline comparisons

There were no significant differences for age (t test) or for gender and autism severity (Chi-square test) between the intervention and control groups at baseline (T1) as shown in Table 2. However, there were baseline differences between groups for SEIFA classification (Chi-square test; p = 0.001). There were no between group differences for the parent or adolescent self-reported outcome measures at baseline (T1).

### Dosage and fidelity

Dosage of the BOOST-A™ intervention was measured by the number of logins to the program and the number of modules completed, obtained through program analytics. Participants in the intervention group logged into the BOOST-A™ an average of five times (range = 0 to 14, *SD* = 3.4). On average, participants completed three of the four modules by T2 (range = 0 to 4; *SD* = 1.1). Participants reported an average of two team meetings at T2 (range = 0 to 5; *SD* = 1.0).

### Intervention effects

AIR There were no significant differences between the intervention and control groups for the primary outcome of overall self-determination as determined by the AIR. The mean (SD) difference in the AIR change score before and after the intervention (i.e. T2 − T1) among parents in the intervention group was 2.3 (8.3) compared to parents in the control group (− 0.2 (7.8); p = 0.13). Similarly, there was no difference in the mean (SD) AIR change score before and after the intervention among the adolescents in the intervention group (6.2 (18.2)) compared to the control group (0.5 (18.9); p = 0.19).

Most outcomes improved over time, with greater improvements for the intervention group, as seen in Table 3. Overall quality of life for the adolescents decreased over the 12 months for both groups, as indicated by the personal well-being index. There were significant between-group differences in three summary score areas favoring the intervention group: career exploration for parents (p = 0.03) and adolescents (p = 0.01); the self-determination sub-scale of Home for parents (p = 0.01); and transition-specific self-determination for parents (p = 0.01). The summary scores for the remaining outcome measures showed no significant differences between groups. Because there was a between-group difference in socio-economic advantage at baseline, a general linear model was used to test whether the differences persisted after adjustment for SEIFA. Results indicated that the significant differences found in career exploration, the self-determination sub-scale of home, and transition-specific self-determination remained after adjustment for SEIFA. Findings from fitting the random effects regression model agreed with the findings drawn from Table 3, and are therefore not shown in detail here.

**Table 3:** Outcomes at baseline (T1) and at 12 months post-intervention (T2)

	Intervention group n = 88^a^		Control group n = 83^a^		Group by time	
	T1 Mean (SD)	Difference T2 − T1 (SD)	T1 Mean (SD)	Difference T2 − T1 (SD)	*T*	*p*
Parent-reported outcomes						
Self-determination (AIR)						
Total	56.6 (9.2)	2.3 (8.3)	58.6 (8.10)	− 0.2 (7.8)	1.52	0.13
Do	14.7 (4.3)	1.1 (3.5)	14.8 (4.0)	0.7 (2.4)	0.61	0.55
School	20.1 (4.6)	0.37 (3.8)	20.4 (4.5)	− 0.3 (3.7)	0.78	0.44
Home	22.8 (3.3)	0.9 (2.2)	23.6 (2.9)	− 0.4 (2.6)	2.59	0.01*
Transition-specific self-determination	75.3 (21.3)	18.9 (19.7)	82.5 (21.3)	8.1 (19.3)	2.68	0.01*
Career planning (CDI-A)	21.5 (8.4)	4.1 (8.8)	21.3 (8.0)	2.6 (7.9)	0.87	0.39
Career exploration (CDI-A)	23.0 (6.2)	3.4 (5.6)	24.7 (6.2)	0.8 (5.6)	2.27	0.03*
Learning climate (LCQ)	4.1 (1.2)	0.4 (0.9)	4.1 (1.0)	0.1 (0.9)	1.79	0.08
Personal wellbeing index (PWI-SC)	63.4 (14.8)	− 0.9 (13.5)	63.3 (12.8)	− 1.1 (11.3)	0.08	0.94
Happiness—life as a whole (PWI-SC)	60.6 (26.3)	3.1 (23.3)	62.0 (22.7)	63.9 (26.0)	0.50	0.62
Adolescent-reported outcomes						
Self-determination (AIR)						
Total	73.7 (21.2)	6.2 (18.2)	76.5 (18.3)	0.5 (18.9)	1.34	0.19
Do	18.0 (4.8)	1.1 (4.0)	18.0 (5.2)	0.4 (5.3)	0.58	0.57
Feel	18.5 (5.4)	0.8 (4.6)	19.2 (5.1)	0.1 (5.6)	0.54	0.59
School	18.9 (6.2)	1.2 (6.8)	17.9 (5.3)	1.4 (4.9)	− 0.13	0.89
Home	21.3 (6.0)	1.2 (5.1)	22.7 (4.9)	− 0.1 (5.9)	1.01	0.32
Transition-specific self-determination	86.0 (23.0)	11.4 (22.7)	90.4 (23.7)	5.2 (21.0)	1.25	0.22
Career planning (CDI-A)	27.9 (10.0)	1.5 (9.6)	30.0 (8.1)	1.8 (8.5)	− 0.11	0.91
Career exploration (CDI-A)	26.5 (7.1)	2.3 (6.4)	28.7 (5.4)	− 1.7 (6.0)	2.78	0.01*
Learning climate (LCQ)	4.6 (1.3)	0.2 (1.1)	4.8 (0.9)	0.0 (1.0)	0.64	0.53
Personal wellbeing index (PWI-SC)	70.8 (20.1)	− 0.7 (18.2)	71.5 (13.8)	− 1.5 (12.9)	0.22	0.83
Happiness—life as a whole (PWI-SC)	67.9 (27.4)	1.0 (25.7)	66.5 (16.4)	4.1 (19.1)	− 0.58	0.56

* Significant difference between Intervention and Control Group; p < 0.05

^a^Intervention group: parent n = 49, adolescent n = 39. Control group: parent n = 45, adolescent n = 38

## Discussion

### Primary outcome: Self-determination

Self-determination was the primary outcome of the study because of the previously established correlation between high levels of self-determination and post-school employment and education [50–52]. There was no change in the total self-determination score found in this study. A potential reason for this may have been the varied levels of adherence to the BOOST-A™, as the average number of modules completed was three indicating many participants did not complete the My Progress module. Another explanation may be the lack of face-to-face training in how to use the BOOST-A™, which was delivered remotely via an online platform. A meta-analysis of the effectiveness of technology-based programs for adolescents on the autism spectrum found that programs that were entirely self-directed by participants had a smaller effect than programs administered by a specialist [32]. Whilst there is a need for programs that are not only effective but also easily accessible, affordable, and user-friendly [32], technology should not be used as a substitute for face-to-face support [53]. Therefore, ensuring direct access to a trained professional to facilitate use of the BOOST-A™ may be an important consideration for future iterations of the program.

A significant difference was found in self-determination between the intervention and control groups was in the Home subscale. This may suggest that the BOOST-A™ supported parents to provide increased opportunities for the adolescent to practice decision-making, goal setting, and problem solving in the home environment. This finding is of interest, given that current literature tends to focus on school as the context to improve adolescents’ self-determination skills, with less focus on the home environment [54]. In addition, the majority of existing transition planning programs focus on supporting school staff to enhance the self-determination of students with disabilities in the school environment [13, 17, 55, 56]. However, parents are possibly the most consistent and enduring influence in their adolescent’s life, especially during the transition from school into post-secondary education or employment [54, 57]. Parents model self-determined behavior in the home environment and provide opportunities for adolescents with autism to make choices; take appropriate risks; and develop skills in problem solving, self-regulation, and assertive communication [58]. A strength of the BOOST-A™ is that it can be used either at school or at home and can be championed by parents and/or teachers.

The increase in opportunities provided at home reflects a potential shift in parents’ expectations for their children, as supported by the results from the process evaluation of the BOOST-A^TM^ [59]. Parents who hold high expectations for their adolescents with autism can increase the adolescent’s self-determined behavior and improve their post-school outcomes [15]. Furthermore, increased frequency of discussions about post-school plans in the home environment has been correlated with increased participation of adolescents on the autism spectrum in transition planning meetings at school [60]. Therefore, changes in the behavior of the parents may result in increased opportunity to engage in transition planning for adolescents on the autism spectrum.

A possible explanation for the observed increase in the Home subscale of self-determination but not the School subscale is that parents were the primary contacts in this trial and the key point of liaison with the research team. Further research in this area might assist in understanding the relationship between the home and school settings, and the opportunities for self-determined behavior provided to adolescents on the autism spectrum in these settings.

### Secondary outcomes

The BOOST-A™ led to a significant increase in career awareness among the adolescent participants. Career awareness is defined as the level of engagement with external sources of career information, such as parents, teachers, and written information, as well as the adolescents attitude towards these sources of information [40]. Career awareness is predictive of being productively engaged in education and employment in the first year out of school [61, 62]. The finding that the BOOST-A™ increased career awareness supports the hypothesis that adolescents on the autism spectrum who use the program may have an increased likelihood of transitioning to post-secondary study and employment after school. No significant differences were found in career planning, which is the amount of planning that has been completed [40]. The lack of significant increases in career planning may have been because not all participants completed the fourth module that supported them to revise goals and progress through planning.

There was also a significant increase in transition-specific self-determination favoring the intervention group. The Transition-specific Self-determination scale looked at the adolescents’ opportunity for active engagement in transition-specific team meetings; exploration of interests and strengths; goal setting; and real-life experiences, such as work experience, mentoring, and part-time work. However, the psychometric properties of the Transition-specific Self-determination scale are currently unknown and so these results should be interpreted with caution. Future research to validate this scale is recommended.

For both groups, adolescent quality of life decreased, whilst happiness with life as a whole increased. The overall reduction in quality of life during adolescence is consistent with a decrease in quality of life that is seen in mid to late adolescence for the general population [63]. This decrease in quality of life is likely because adolescence is a period in which young adults experience a shift in roles and seek greater independence, which is often at odds with their continued dependence on caregivers [63]. In addition, adolescents are presented with many new challenges as they transition out of high school that are likely to impact on quality of life. Overall quality of life for both groups was below the normative range for Australia, which is between 73.4 and 76.4 points out of 100 [42]. Evidence suggests that quality of life among people on the autism spectrum is lower than that of people without autism across the lifespan [7]. Therefore, further research is warranted that looks at quality of life during the transition period for adolescents on the autism spectrum, and how this compares to adolescents without autism.

There appeared to be a discrepancy in that overall quality of life decreased whilst happiness with life as a whole increased. One possible explanation is provided by the concept of the ‘just right challenge’ in self-determined learning theory, which describes how opportunities should provide an optimal level of challenge to enhance adolescents' capacity to regulate their feelings and actions [64]. Whilst encountering challenges in the transition planning period, adolescents may describe a reduction in overall their quality of life but an increase in happiness as they learn new skills and overcome challenges. Parents in this study rated their adolescent’s quality of life lower than the adolescents’ self-ratings. This finding is consistent with previous research that proposed adolescents on the autism spectrum may perceive the difficulties they face to be less of a problem than their parents [7]. In addition, parents may have made assumptions on the meaning of a good life without asking their children what would make them happy, which is an important prerequisite for emotional wellbeing [65]. Discrepancies between parent and adolescent perspectives of quality of life is an issue requiring further exploration in future research.

Overall, only one area showed a significant difference as reported by the adolescents, in comparison to three areas as reported by parents. The lack of significant differences as reported by adolescents is noteworthy, because the BOOST-A^TM^ aimed to improve adolescents’ perceived autonomy and control, as this has been linked to improved post-school outcomes [66], and increased subjective quality of life [63]. This indicates more work may need to be done to improve adolescent outcomes in the transition planning process using the BOOST-A™.

The lack of between-group differences for many of the outcomes suggests that the BOOST-A™ was more helpful for some adolescents on the autism spectrum than it was for others. This finding may be due to the range of characteristics of people on the autism spectrum and is consistent with evidence that there is a wide variability in outcomes for children on the autism spectrum [67, 68]. Another potential reason for the varied results for participants could be that some had comorbid diagnoses such as attention deficit hyperactivity disorder and anxiety, which could have introduced additional considerations related to transition planning. A process evaluation was performed immediately following the quasi-randomized controlled trial to determine individual characteristics and contextual factors that support positive outcomes from using the BOOST-A™. The process evaluation found that whilst the BOOST-A™ supported some adolescents to engage in the transition planning process and develop new insights that led to clearer plans for the future, barriers included not having access to a professional to guide the way and difficulty motivating the adolescent to engage in the process [59]. Full results of the evaluation are reported separately [59].

### Limitations

A limitation of this study was that the participating adolescents’ autism diagnosis was based on parent-report and confirmed by the SRS-2 [35]. Ideally, the Autism Diagnostic Observation Scale (ADOS) [69] would have been used to verify autism diagnosis, since it has good sensitivity and specificity [70]. However, this was not possible because the ADOS is administered face-to-face and study participants came from a wide spread of geographic locations across Australia. The study had a low attrition rate for parents (9% control; 12% intervention), but higher for the adolescents (10% control; 31% intervention). Consequently, the final analysis was based on a sample containing less than 40 adolescents in each group, which may have resulted in the study being underpowered to detect between-group differences for all the outcomes measured.

Use of a quasi-randomized and non-blinded treatment allocation presented potential sources of bias. However, baseline comparisons revealed no significant differences between the control and intervention groups in outcomes and demographic variables other than socio-economic status. A general linear model confirmed that the significant between-group differences for the intervention effects remained after adjustment for socio-economic status. Given the difference in socio-economic status, it would have been optimal to have collected information about parents’ academic qualifications and professions to determine if there between-group differences in these areas. Although participants were excluded if they were participating in any other formalized transition planning program, it would have been optimal to gather data from the control group about any informal transition planning that may have been initiated by parents or schools during the study period. Different types of regular practice undertaken by the control group may have influenced their outcomes, so this study may have underestimated the true impact of BOOST-A™ over a standardized control group with only basic transition planning.

The timeframes for this study did not allow for follow-up to determine whether the effects of the intervention were maintained or to gather information about participants’ employment outcomes after graduation from school. This is a limitation because career readiness outcomes, such as self-determination, that were used in the study are only correlated with employment. Their observed improvement in this study may not necessarily lead to an increase in employment [71]. The use of employment as an outcome would have reduced the risk of bias introduced by the use of self-report measures, such as social desirability. It is recommended that future studies are of sufficient duration to explore the maintenance of the changes in career exploration and self-determination over time, as well as post-school employment outcomes after using the BOOST-A™.

Strengths of the current study included the use of multiple raters (parents and adolescents), blinding of participants to trial hypothesis, as well as self-report measures that eliminated the need for blinding of evaluators. The inclusion of a control group in the study ensured maturation bias did not influence results, especially given the 12-month duration of the study.

## Conclusion

This study found that there were no significant differences between groups for the primary outcome of overall self-determination. There is preliminary evidence that the BOOST-A™ is effective in increasing career exploration and opportunities for self-determination in the home environment for adolescents on the autism spectrum.

## Abbreviations

BOOST-A™: Better OutcOmes & Successful Transitions for Autism
ID: intellectual disability
AIR: AIR Self-Determination Scale
CDI-A: Career Development Inventory—Australia—Short Form
PWI-SC: personal wellbeing index-school
LCQ: learning climate questionnaire
SEIFA: Socio-Economic Indexes for Areas
